# Enhanced Optical Properties of Germanate and Tellurite Glasses Containing Metal or Semiconductor Nanoparticles

**DOI:** 10.1155/2013/385193

**Published:** 2013-04-22

**Authors:** Cid Bartolomeu de Araujo, Diego Silvério da Silva, Thiago Alexandre Alves de Assumpção, Luciana Reyes Pires Kassab, Davinson Mariano da Silva

**Affiliations:** ^1^Departamento de Física, Universidade Federal de Pernambuco, 50740-540 Recife, PE, Brazil; ^2^Departamento de Engenharia de Sistemas Eletrônicos, Escola Politécnica da USP, 05508-900 São Paulo, SP, Brazil; ^3^Laboratório de Tecnologia em Materiais Fotônicos e Optoeletrônicos, Faculdade de Tecnologia de São Paulo, CEETEPS/UNESP, 01124-060 São Paulo, SP, Brazil

## Abstract

Germanium- and tellurium-based glasses have been largely studied due to their recognized potential for photonics. In this paper, we review our recent studies that include the investigation of the Stokes and anti-Stokes photoluminescence (PL) in different glass systems containing metallic and semiconductor nanoparticles (NPs). In the case of the samples with metallic NPs, the enhanced PL was attributed to the increased local field on the rare-earth ions located in the proximity of the NPs and/or the energy transfer from the metallic NPs to the rare-earth ions. For the glasses containing silicon NPs, the PL enhancement was mainly due to the energy transfer from the NPs to the Er^3+^ ions. The nonlinear (NL) optical properties of PbO-GeO_2_ films containing gold NPs were also investigated. The experiments in the pico- and subpicosecond regimes revealed enhanced values of the NL refractive indices and large NL absorption coefficients in comparison with the films without gold NPs. The reported experiments demonstrate that germanate and tellurite glasses, having appropriate rare-earth ions doping and NPs concentration, are strong candidates for PL-based devices, all-optical switches, and optical limiting.

## 1. Introduction

Suitable composites for photonic applications have to present large transmittance, high refractive index, low cut-off phonon energy, and large nonlinear optical response. Germanate and tellurite glasses have these characteristics and were identified as good hosts for trivalent rare-earth ions (REI) and metal or semiconductor nanoparticles (NPs) [1–12]. In these glasses, the linear and nonlinear optical properties may be largely enhanced due to the presence of the NPs. Specifically, in glasses containing metal NPs when the incident light or the photoluminescence (PL) wavelengths are near the localized surface plasmon resonance (LSPR) wavelength, *λ*_SP_, a large PL enhancement may occur. In principle, *λ*_SP_ depends on the host and metal dielectric functions as well as on the dimensions and shape of the NPs. The PL efficiency of a composite for a given incident wavelength depends on the LSPR, the NPs concentration, and the density of phonon states in the host material. In particular, REI doped glasses containing metallic NPs have been investigated because their luminescence may be intensified by energy transfer from the metallic NPs and/or due to enhancement of the local field that acts on the REI located in the proximity of the NPs [10, 11]. On the other hand, glasses containing silicon NPs (semiconductor quantum dots) may act as efficient absorbers, and the energy stored in the Si NPs may be transferred to REI, and thus they may contribute for PL enhancement in various wavelengths ranges that can be selected controlling the quantum dots sizes. Some evidence of this effect was reported long ago for silicon-rich silica glass [13–16]. 

The first report on nucleation of silver NPs in germanate glasses (PbO-GeO_2_) was presented in [17], whereas the possibility of production of silver NPs in tellurite glasses was published for the first time in [18]. Also it was reported for the first time the nucleation of copper NPs in PbO-GeO_2_ thin films for applications in all-optical switching [19]. The various studies reported for germanate and tellurite glasses, with metallic NPs and different REI, demonstrated the large potential of these materials for photonic applications. On the other hand, the influence of Si NPs on the PL properties of glasses remains a unexploited subject. Recently, we developed a procedure to nucleate Si NPs in germanate glasses that was very efficient to improve the PL properties of germanate glasses.

In this paper, we review recent experiments based on the nucleation of Ag, Au, and Si NPs in germanate and tellurite bulk glasses containing REI and discuss the optical behavior of the samples under different excitation conditions. Besides the work with bulk samples, the production of germanate thin films using the radio-frequency sputtering technique is also reported. The nucleation of gold NPs in the films is described, and a study of the NPs influence on the films' nonlinear optical properties is presented. 

The paper is organized as follows. In Section 2, we describe the fabrication methods and the characterization techniques used to study the samples. In Section 3, the structural characteristics of the samples as well as their optical characteristics are discussed. Finally in Section 4, we comment on the perspectives of applications for the kind of composites discussed here.

## 2. Methods

### 2.1. Production of the Bulk Samples

Germanate (GeO_2_-PbO and GeO_2_-Bi_2_O_3_) and telurite (TeO_2_-ZnO and TeO_2_-WO_3_-Bi_2_O_3_) glasses were prepared with high-purity reagents (99.999%) using the melt-quenching method followed by adequate heat treatment (HT) that depends on the transition temperature of each glass composition. The compositions studied are shown in Table 1 as well as the parameters used in the fabrication process.

The reagents were melted in crucibles made of platinum (for the tellurite glasses) or alumina (for the germanates). Mechanical stirring during the melting was applied to germanate glasses, to optimize transparency and homogeneity. After cooling to room temperature inside the furnace, the samples were polished, cut, and then submitted to additional HT to nucleate metallic NPs (metallic ions Ag^+^ and Au^+^ from AgNO_3_ and Au_2_O_3_ are reduced to Ag^0^ and Au^0^, resp., for nucleation of the NPs). Samples without metallic NPs were also fabricated to be used as reference.

The melt-quenching technique was also used for the fabrication of the samples with silicon quantum dots mixing Si nanograins having average diameter of 100 nm with the base germanate glass constituents. The starting reagents were melted inside an alumina crucible for 1 h at 1200°C, quenched in air in a preheated brass mold, and annealed at 420°C to minimize internal stress. Finally, the glasses were cooled to room temperature inside the furnace. After cooling, the samples' surfaces were polished to optical quality. Samples without Si-NCs and doped with Er^3+^ were also fabricated to be used as reference. Transparent glasses stable against crystallization were produced. The density of Si-NCs was controlled by heat-treating the samples for time intervals of 3, 48, and 72 hours, at 420°C. 

### 2.2. Production of the Thin Film Samples

The production of noncrystalline films with and without gold NPs was made by RF sputtering (at 13.56 MHz) under 5.0 mTorr of argon. For the glass target preparation, the oxide powders were mixed and then submitted to 8 tons uniaxial press. Then, sinterization at 750°C, for 10 h, was performed followed by HT at 370°C, at room atmosphere. The films without NPs were prepared using a target with the composition 40GeO_2_-60PbO (in wt%). The films with gold NPs were prepared using the same glass target and a metallic gold target (purity of 99.99%). The two targets were simultaneously sputtered. The films were deposited on silica substrates placed 15 cm far from the targets. The RF power applied in each target was 50 W for the GeO_2_-PbO target and 6 W for the gold target; the deposition time was set to obtain films with thickness of *≈*1.0 *μ*m. The HT of the films was performed during different times and temperatures to nucleate the gold NPs. 

### 2.3. Techniques Employed for Characterization of the Samples

To investigate the size and shape of the NPs, a Transmission Electron Microscope (TEM) operating at 200 kV and a high-resolution transmission electron microscope (HR-TEM) operating at 300 kV were used. electron diffraction measurements were performed to confirm the crystalline structure of the NPs. 

All optical measurements were made at room temperature.

A spectrophotometer that operates in the visible and near-infrared regions was used to measure the absorption spectra of the samples.

PL spectra were measured with different kinds of excitation sources, depending on the sample. In the case of the Eu^3+^ doped samples, excitation at 405 nm was obtained from a 15 W xenon lamp followed by a 0.25 m monochromator equipped with a holographic grating and the signals were analyzed by a phase fluorometer. For the Er^3+^ doped samples, a CW 980 nm diode laser and an ytterbium laser operating at 1050 nm were used. For the Tm^3+^ doped samples, an Nd^3+^: YVO laser operating at 1047 nm was utilized the acquisition of signals was obtained with a monochromator with a photomultiplier connected to a lock-in and a computer. 

The nonlinear measurements at 532 nm were made using the second harmonic of a Nd: YAG laser (Q-switched and mode-locked), coupled to a pulse selector for experiments with single pulses of 80 ps at 7 Hz. The Z-scan technique [20] was used to measure the nonlinear refractive index and nonlinear absorption coefficient of the samples. The response time of the nonlinearity was determined by a Kerr gate setup [21] based on a Ti-sapphire laser (800 nm; 76 MHz; 150 fs) as the light source. The signals were processed using a boxcar and a computer.

## 3. Results

### 3.1. Luminescence of Bulk PGO-Eu:Ag and BGO-Eu:Au

**Figure 1:**
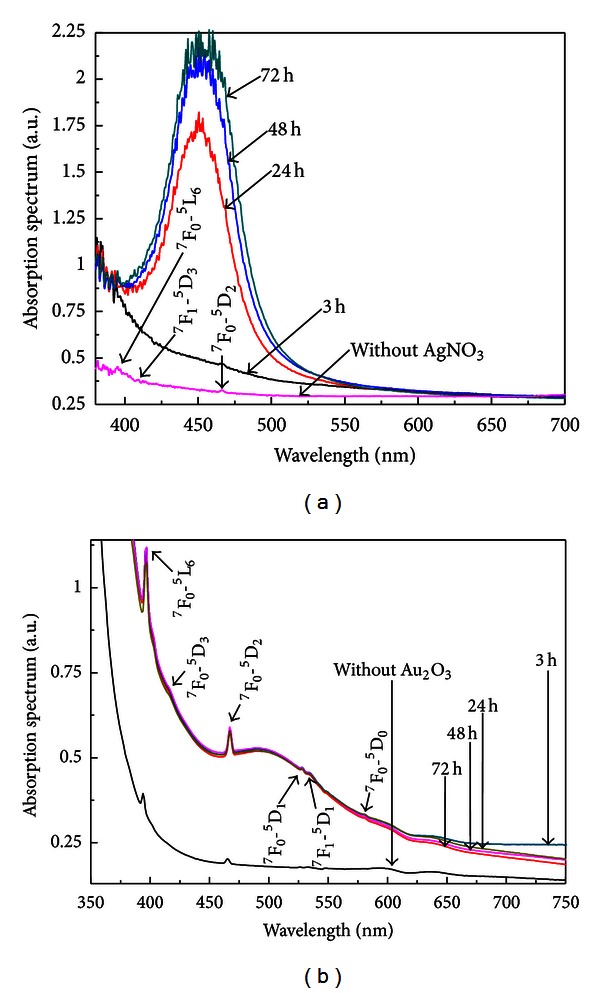
Absorption spectra of the Eu^3+^ doped samples heat-treated during different times. (a) PGO-Eu:Ag. (b) BGO-Eu:Au. Also presented are the spectra of both glasses without metallic NPs [1].

The influence of the metallic NPs on electric-dipole and magnetic-dipole transitions of Eu^3+^ doped germanate glasses was studied in PGO-Eu:Au and BGO-Eu:Au samples with various NPs concentrations and their absorption spectra are shown in the Figures 1(a) and 1(b).

An absorption band in the blue-yellow region is a strong evidence of the nucleation of a large density of metallic NPs. In Figure 1(a) the band centered at *≈*450 nm is attributed to the LSPR of the silver NPs. Notice that the band amplitude increases for longer HT times, indicating that the density of NPs is also increasing.  Figure 1(b) exhibits a broad absorption band centered at ≈500 nm and a background that extends toward the near infrared, due to the LSPR of the gold NPs and their aggregates. 

**Figure 2:**
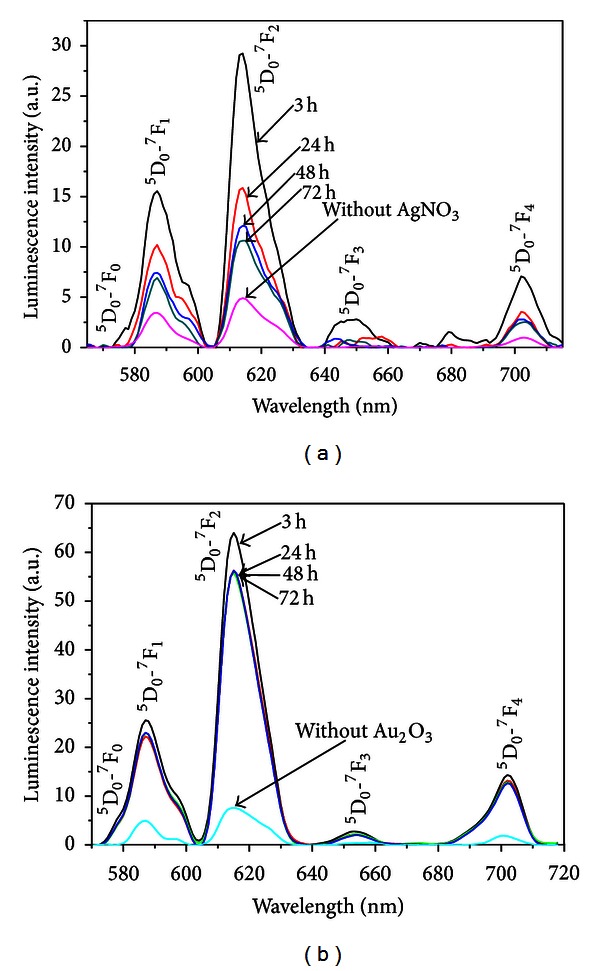
Luminescence spectra of the Eu^3+^ doped samples heat-treated for different times. (a) PGO-Eu:Ag. (b) BGO-Eu:Au. Also presented are the spectra of both glasses without metallic NPs. Excitation wavelength: 405 nm [1].

PL spectra of the same samples for excitation at 405 nm are shown in the Figures 2(a) and 2(b).

The spectra consist of the 4*f-*4*f *transitions associated to the Eu^3+^ ions: ^5^D_0_-^7^F_1_ (585 nm), ^5^D_0_-^7^F_2_ (614 nm), ^5^D_0_-^7^F_3_ (650 nm), and ^5^D_0_-^7^F_4_ (700 nm). The results indicate that the PL amplitudes are affected by the presence of the metallic NPs, for both samples.

It is clearly noticed that the samples with either silver or gold NPs reach maximum PL enhancement for HT during 3 hours; for longer HT times, it is observed the quenching of the PL spectra for both samples. This behavior is understood considering that with the increasing of the HT time, more Eu^3+^ ions become so near from the NPs that energy transfer occurs from the directly excited ions to the NPs. The quenching phenomenon occurs because of short-distance Eu^3+^-NPs the dipole-dipole interaction between the ion and an NP becomes large [22]. 

**Figure 3:**
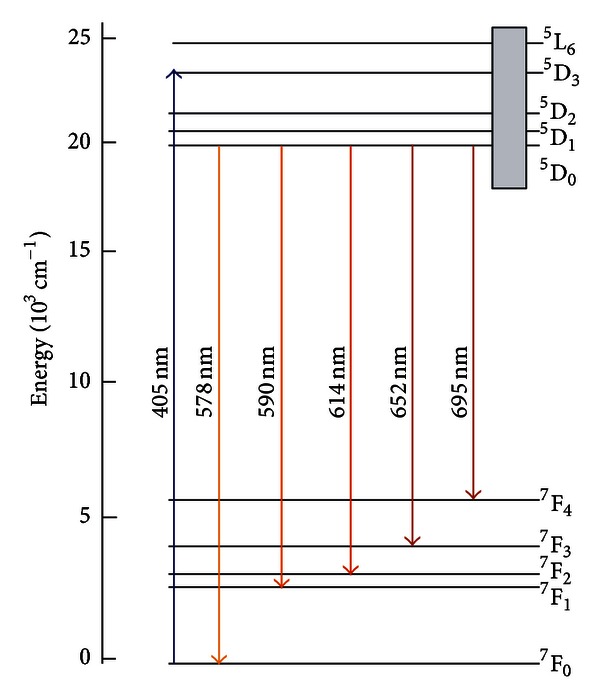
Simplified energy levels scheme of Eu^3+^ ions with indication of the radiative transitions observed. The shaded area indicates the position of the localized surface plasmon band [1].

A simplified energy diagram of the Eu^3+^ ion is shown in Figure 3. Notice that the excitation and the PL wavelengths are near resonance with the LSPR.

**Figure 4:**
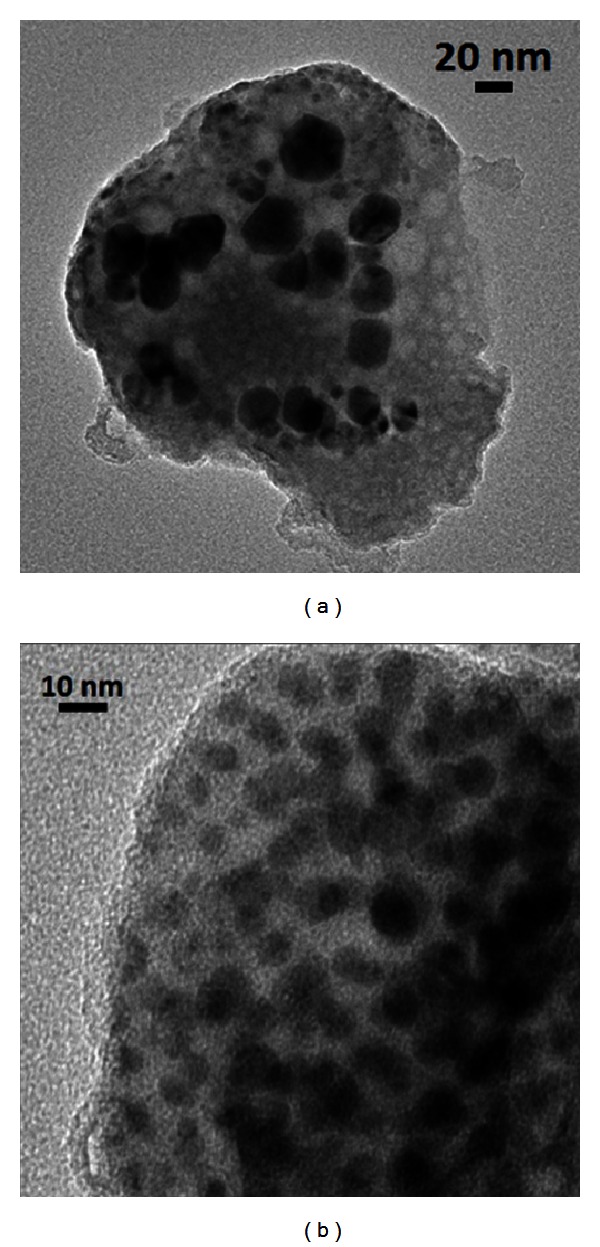
TEM image of the Eu^3+^ doped samples: (a) GeO_2_-PbO glass with silver NPs heat-treated for 72 hours, (b) GeO_2_-Bi_2_O_3_ glass with gold NPs heat-treated for 24 hours [1].

Figure 4 shows the TEM images of both samples with a distribution of nearly spherical NPs that contributes to the relatively narrow LSPR bands observed in the absorbance spectra.

In conclusion, we emphasize that nucleation of large concentrations of silver or gold NPs was obtained in Eu^3+^ doped germanate glasses as illustrated by the strong absorption band associated to the LSPR. Notice that the presence of silver or gold NPs in the glasses produces a large PL enhancement. Our results are understood considering the energy transfer process from the metallic NPs to the ions and the enhancement of the confined electromagnetic field in the vicinity of the NPs. The PL intensity enhancement associated to magnetic dipole transitions (^5^D_0_-^7^F_1_) of Eu^3+^ ions are due to the confined optical magnetic field. Comparisons of the results for the mixed electric-dipole magnetic-dipole transition (^5^D_0_-^7^F_3_) with the relative growth of the ^5^D_0_-^7^F_2_ transition indicate that the influence of optical magnetic field is relevant also in this case. In other words, although the magnetic response at optical frequencies is usually weak, the location of europium ions in a region where the electromagnetic field is confined contributes for the increase in the signals associated to magnetic dipole transitions. Furthermore, the 1000% enhancement of the PL corresponding to transition ^5^D_0_-^7^F_2_ is a remarkable result [1].

### 3.2. Energy Transfer and Frequency Upconversion in Bulk PGO-Er/Yb:Ag

**Figure 5:**
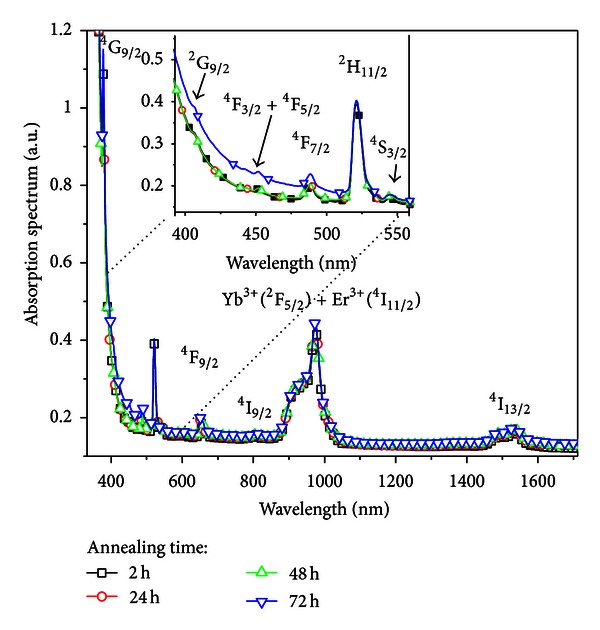
Absorption spectra of PGO-Er/Yb:Ag glasses containing silver NPs for different heat-treatment times [2].

**Figure 6:**
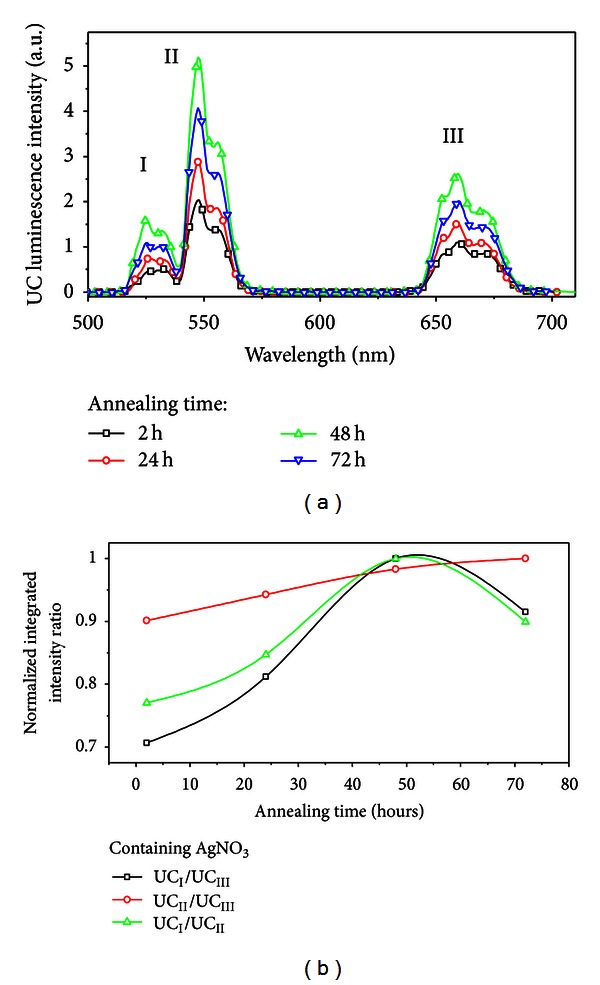
(a) Frequency upconversion spectra of the of Er^3+^/Yb^3+^ codoped PbO-GeO_2_ glasses containing silver NPs for different heat-treatment times. (b) Normalized integrated upconversion intensity (UC_I_: peak centered at 525 nm, UC_II_: peak centered at 550 nm, and UC_III_: peak centered at 662 nm) as a function of the annealing time [2].

The energy transfer and frequency upconversion processes in Yb^3+^/Er^3+^ doped PbO-GeO_2_ glass were studied in the samples containing silver NPs. The excitation wavelength was 980 nm in resonance with the Yb^3+^ transition ^2^F_7/2_-^2^F_5/2_. The efficient energy transfer from resonantly excited Yb^3+^ to Er^3+^ and the influence of the Ag NPs contributed to large enhancement of the whole upconverted spectrum. Absorption and emission spectra of the PGO-Er/Yb:Ag samples for different HT times are shown in Figures 5 and 6(a) respectively. Emission bands centered at 525, 550, and 662 nm that correspond, respectively, to the transitions ^2^H_11/2_→^4^I_15/2_, ^4^S_3/2_→^4^I_15/2_, and ^4^F_9/2_→^4^I_15/2_ can be seen in Figure 6(a).  Figure 6(b) shows that the relative intensity of the upconversion bands can be adjusted by an appropriate choice of the HT time that controls the amount of silver NPs formed in the sample. 

**Figure 7:**
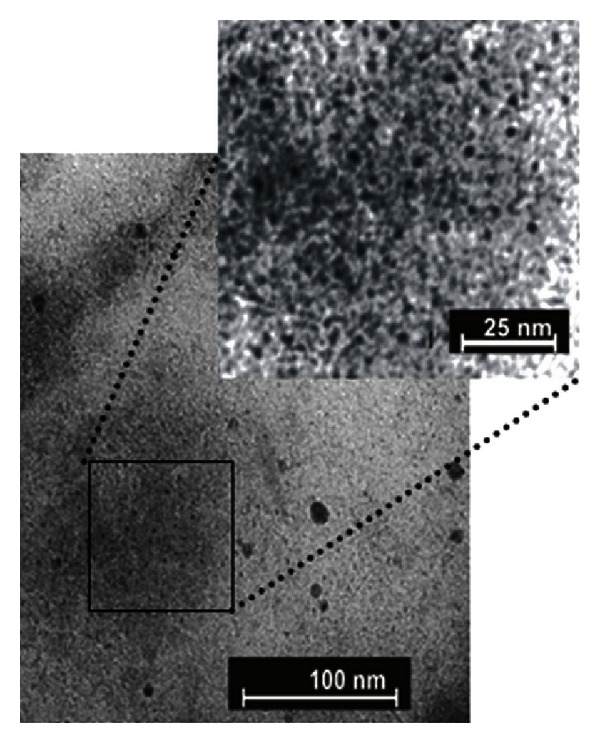
TEM images of the samples annealed during 48 h at 420°C [2].

The LSPR band is not observed in Figure 5 because the amount of silver NPs is not enough to originate a strong band. However, we estimate, with basis on the dielectric function of silver [19], that *λ*_SP_ is located between *≈*400 and *≈*500 nm, as shown in the Figure 1(a). However, the presence of silver NPs in the samples is confirmed by the TEM images shown in Figure 7. 

**Figure 8:**
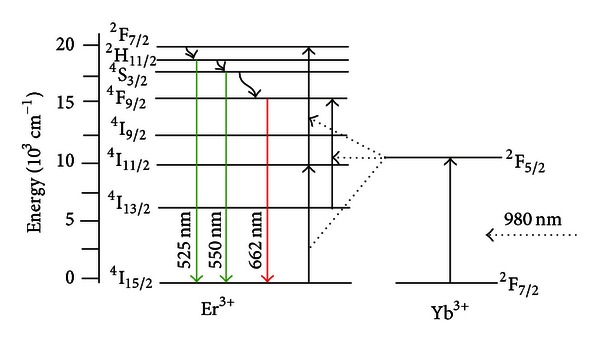
Energy level diagram of Er^3+^ and Yb^3+^ ions illustrating possible upconversion pathways for Er^3+^/Yb^3+^codoped glasses. The solid straight lines with upward and down arrows indicate optical transitions; dotted lines and wavy arrows denote ET processes and non-radiative relaxation, respectively [2].

The emission peaks observed in the spectra of the Figure 6(a) are due the transitions indicated in the simplified energy level diagram for the Er^3+^ and the Yb^3+^ ions in Figure 8. The proximity between the green PL wavelengths and the LSPR favors the PL intensity enhancement. The red emission is also intensified for larger values of annealing times and this is attributed to the nucleation of larger silver NPs and aggregates.

The ratio between the integrated intensities of transitions centered at 525 nm and 550 nm changes from 0.77 (HT *≈* 0 h) to *≈*1.0 (HT *≈* 50 h) because the transition at 525 nm is closest to the estimated LSPR wavelength than 550 nm.

In summary, with the present results we demonstrated the simultaneous exploitation of the enhanced local field contribution due to silver NPs and energy transfer processes between two different RE ions in order to control the PL spectrum of the glassy composite material. This approach can be applied for different RE ions in order to improve the efficiency of luminescent displays.

### 3.3. Frequency Upconversion in Bulk TZO-Tm:Ag and TWB-Er:Ag

**Figure 9:**
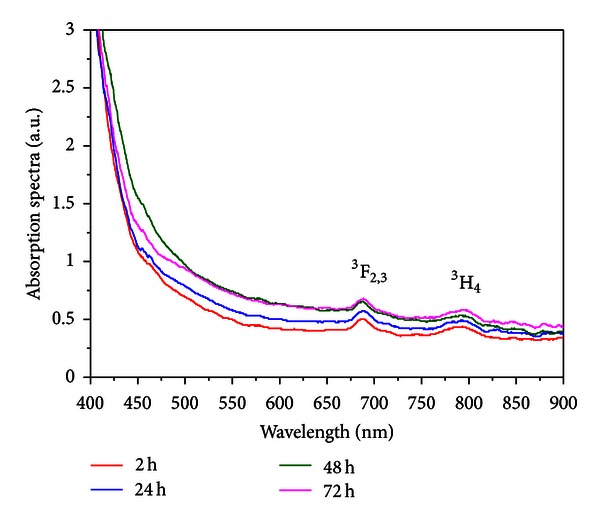
Absorption spectra of TZO-Tm:Ag glasses for different heat-treatment times [3].

**Figure 10:**
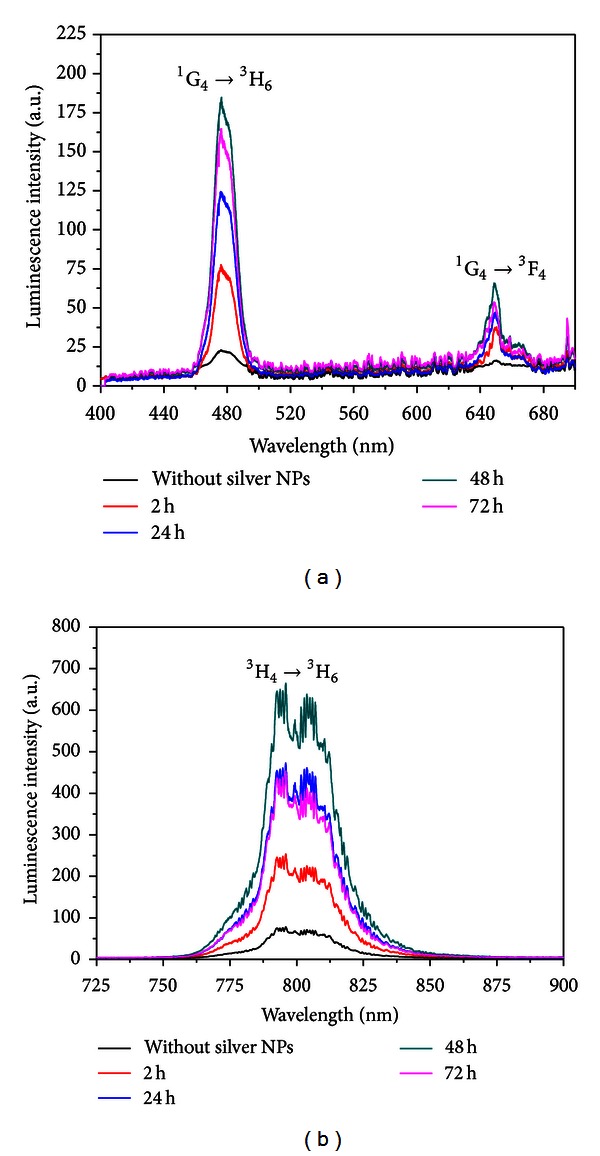
Emission spectra of TZO-Tm:Ag glasses for different heat-treatment times [3].

The frequency upconversion properties of Tm^3+^ doped TeO_2_-ZnO glasses containing silver nanoparticles was reported for the first time in [3]. For excitation, we used a laser operating at 1050 nm. The influence of the silver NPs in the infrared-to-visible and infrared-to-infrared upconversion processes associated to the Tm^3+^ ions was studied. Absorption and emission spectra of the TZO-Tm:Ag samples heat-treated for different HT times are shown in Figures 9 and 10, respectively.

**Figure 11:**
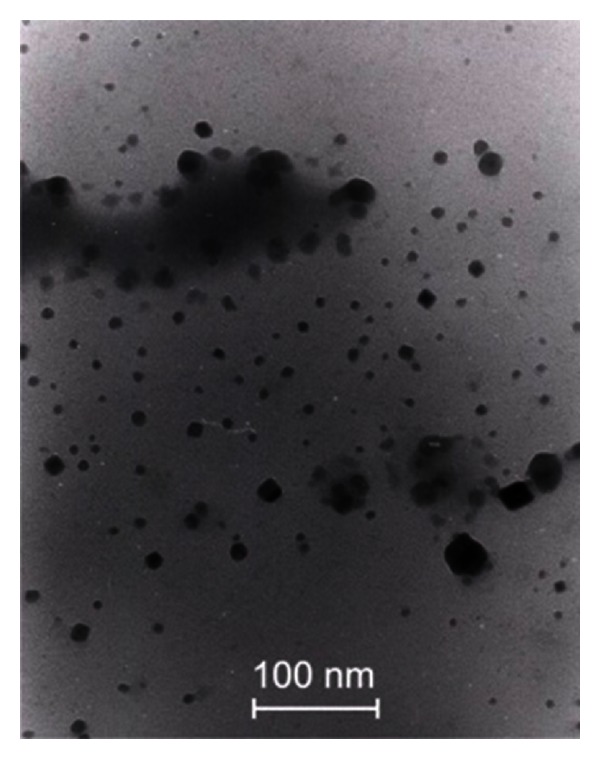
TEM images of the samples heat-treated during 48 h [3].

As in the previous case (Section 3.2), the LSPR band is not observed because the amount of NPs is not enough to show a noticeable band. We estimated that the *λ*_SP_ of isolated NPs to be located in the range 400 to 500 nm because PGO and TZO have dielectric functions of approximate values; the presence of silver NPs is confirmed by the TEM image presented in Figure 11. 

**Figure 12:**
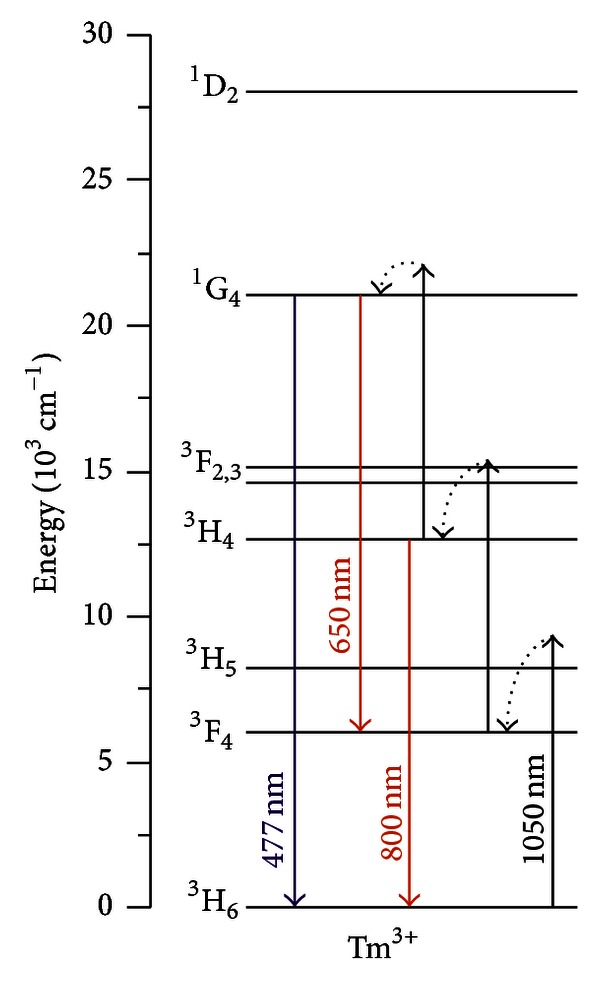
Simplified energy level diagram of Tm^3+^ ions with indication of the upconversion pathways and wavelengths [3].

Figure 12 presents a simplified energy diagram for the Tm^3+^ ions with indication of the excitation pathways and the PL transitions observed. 

One order of magnitude enhancement is observed for the whole PL spectra that is a remarkable result. The spectra corresponding to HT during 72 hours show partial quenching of the PL intensity as reported for other samples.

The dependence of the upconversion signals with the laser intensity was analyzed to identify the routes corresponding to each upconversion emission. The log-log plots of the upconversion intensities corresponding to transitions ^1^G_4_→^3^H_6_, ^1^G_4_→^3^F_4_ and ^3^H_4_→^3^H_6_ present slopes of *≈*2.7, *≈*2.8, and *≈*1.8, respectively. These results indicate that the PL bands at 477 nm and 650 nm are due to the absorption of three laser photons, while the transition ^3^H_4 _→^3^H_6_ is due to the absorption of two photons. Considering the mismatch between the incident photon energy and the energy levels, we conclude that the upconversion processes occur because the intermediate steps are phonon-assisted. Even the excited state absorption ^3^F_4_→^3^F_2,3_ which is resonant is followed by emission of phonons due to the decay from level ^3^F_2,3_ to the level ^3^H_4_. 

**Figure 13:**
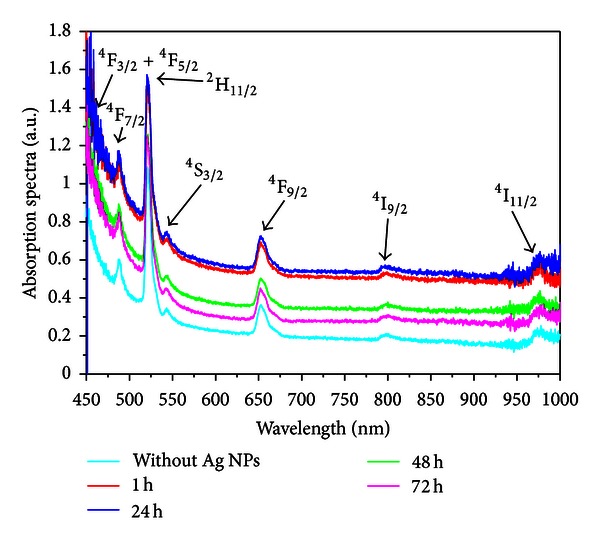
Absorption spectra of TBW-Er:Ag glasses for different heat-treatment times [4].

**Figure 14:**
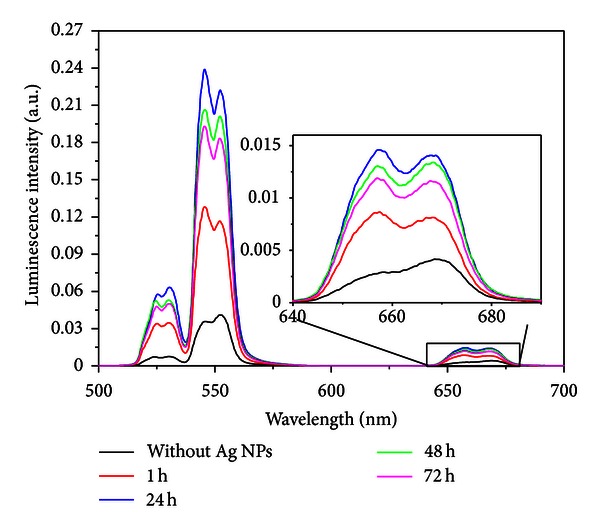
Emission spectra of TBW-Er:Ag glasses for different heat-treatment times [4].

**Figure 15:**
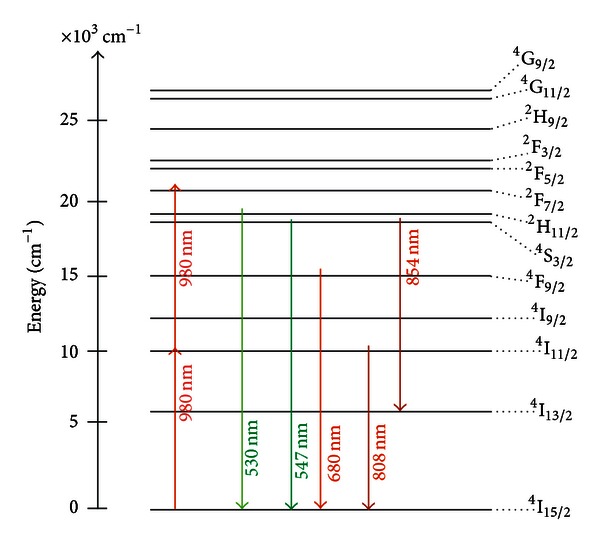
Simplified Er energy levels diagram illustrating theupconversion process by energy transfer and by excited state absorption.

The influence of the Ag NPs on the upconversion emission of the TBW-Er:Ag glass was investigated using a laser operating at 980 nm in resonance with transition ^4^I_15/2_-^4^I_9/2_ [4]. Absorption and PL spectra of the samples heat-treated for different time intervals are shown in Figures 13 and 14, respectively. A simplified Er^3+^ energy diagram showing possible process related to the absorption of the laser energy is shown in Figure 15.

**Figure 16:**
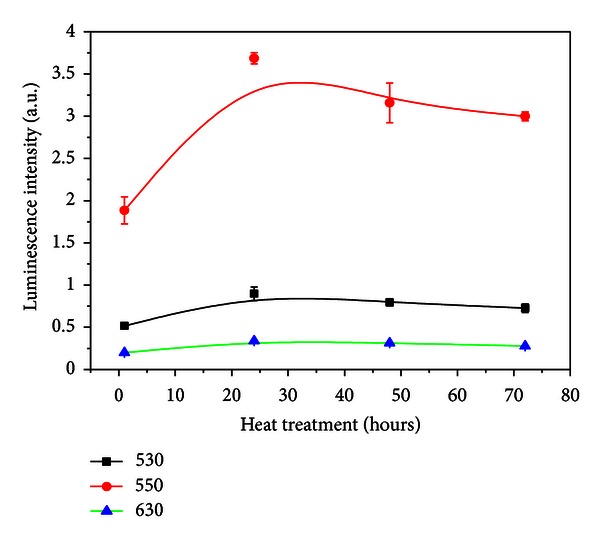
Dependence of the upconverted signals on the heat treatment corresponding to ^2^H_11/2_→^4^I_15/2_ and ^4^F_9/2_→^4^I_15/2_ transitions [4].

By comparing the emission spectra of the sample without silver NPs with the sample heat-treated during 24 h it can be seen that the ^2^H_11/2_→^4^I_15/2_, ^4^S_3/2_→ 2 ^4^I_15/2_ and ^4^F_9/2_→^4^I_15/2_ transitions increased by *≈*700%. For HT during 48 h and 72 h, quenching of the PL is observed. The dependence of the upconverted intensities exhibited a quadratic dependence with the laser intensity and the dependence with the HT time is shown in Figure 16. 

**Figure 17:**
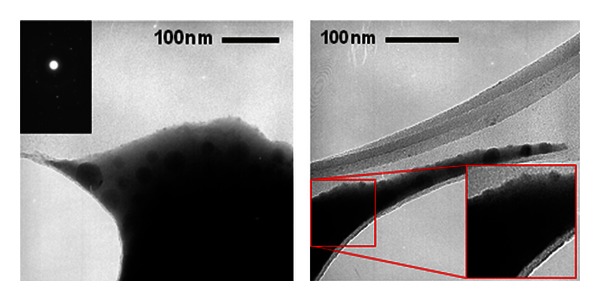
Transmission electron microscope images of TBW-Er:Ag sample with silver NPs after heat-treatment of 24 h [4].

Figure 17 shows TEM images of a sample after HT during 24 h. The black spots are due to the silver NPs which have average size of about 40 nm. A small amount of silver NPs was observed with average diameter of *≈*10 nm. The electron diffraction pattern is also shown in the inset.

The results presented in this section demonstrate that the approach of increasing PL of rare-earth doped tellurite glasses by nucleation of metallic NPs can be successful also for this glass family that has proved already its importance for photonic applications.

### 3.4. Stokes and Antistokes Luminescence of BGO-Er:Si

**Figure 18:**
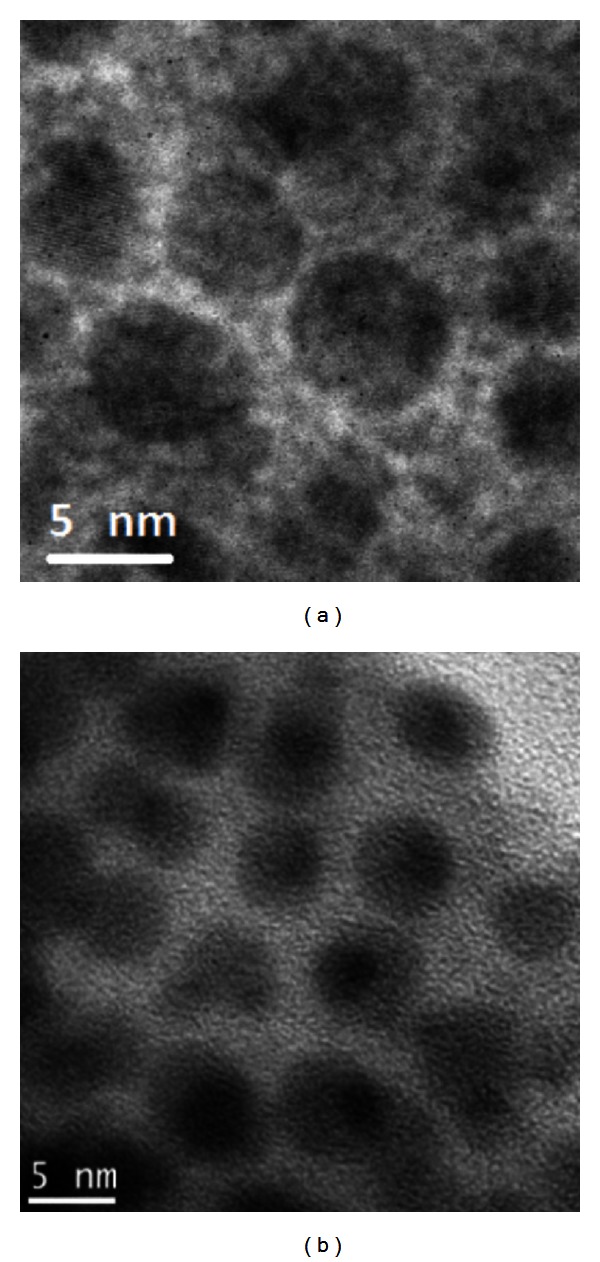
TEM images of Si NPs in Er^3+^ doped GeO_2_-Bi_2_O_3_ glasses heat-treated during 3 h (a) and during 72 h (b) [5].

The first observation of PL enhancement in BGO-Er:Si excited at 980 nm was reported in [5]. The samples fabricated presented large concentration of Si NPs with sizes varying from 2 to 10 nm as it is illustrated in Figure 18 for the samples heat-treated during 3 h and 72 h.

**Figure 19:**
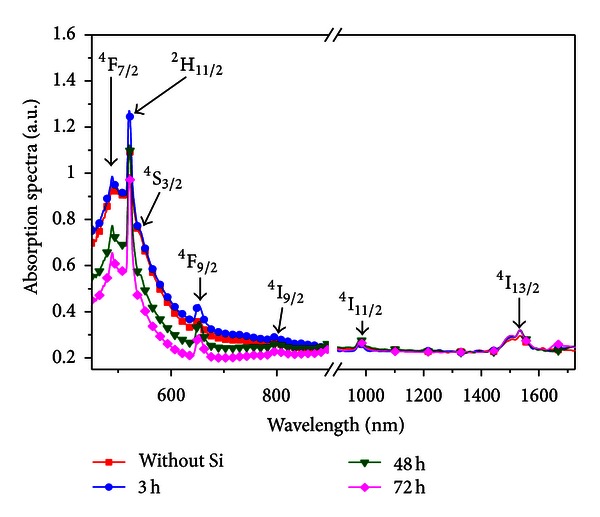
Absorption spectra of Er^3+^ doped GeO_2_-Bi_2_O_3_ glass heat-treated during different time intervals to nucleate Si-NPs. The spectrum of a sample without NPs is shown for reference [5].

**Figure 20:**
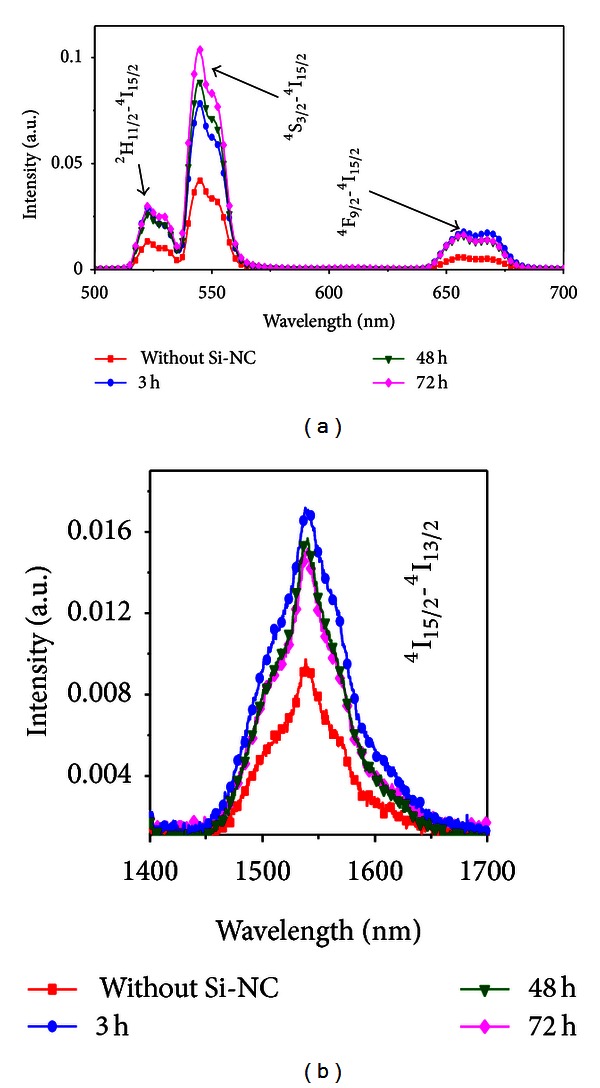
Emission spectra of the Er^3+^ doped GeO_2_-Bi_2_O_3_ glass with Si NPs heat-treated during different time intervals. (a) Infrared-to-visible frequency upconversion. (b) Frequency downconversion [5].

Figures 19 and 20 present absorption and emission spectra of the samples studied including results for a sample without Si NPs used as reference sample. The PL signals were obtained by excitation of transition ^4^I_15/2_-^4^I_9/2_.

Absorption bands associated with Er^3+^ ions are observed as well as a broad absorption band in the range 450–550 nm that was ascribed to the electronic transition ^2^P_1/2_→^2^P_3/2_ of Bi^2+^ ions, according to the literature [23, 24]. A partial quenching of the Er^3+^ transitions ^4^I_15/2_→^2^H_11/2_ and ^4^I_15/2_→^4^F_7/2_ due to the presence of the Bi^2+^ ions can be observed. However, the quenching is reduced in the samples heat-treated during longer times. The PL band in the infrared region, centered at 1530 nm, is due to the ^4^I_13/2_→^4^I_15/2_ transition. The Er^3+^ transitions that correspond to all PL bands are indicated in Figure 15. Note that the signal at 545 nm is enhanced by *≈*200% for the sample heat-treated during 72 hours. For the other emissions centered at 525 nm, 660 nm, and 1530 nm, we observed *≈*100% enhancement in the PL amplitudes for the samples heat-treated during 72 hours. In addition, although the PL spectrum becomes stronger for longer heat-treatment time, the bands' profiles do not change. These results indicate that a large fraction of the Er^3+^ ions are located near the interfaces between the Si NPs and the germanate matrix. This is in accordance with [25] that showed a low solubility of Er^3+^ in Si.

**Figure 21:**
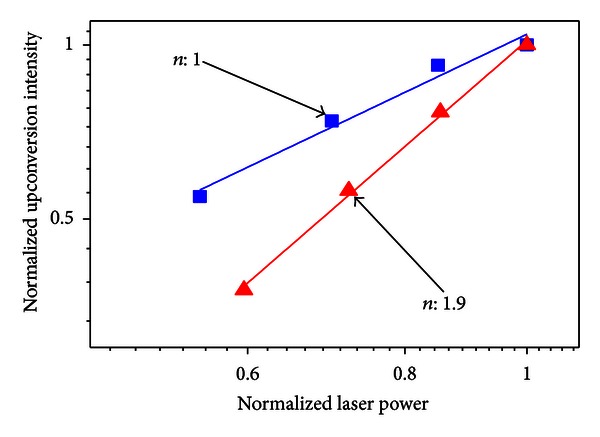
Integrated luminescence intensity as a function of the laser intensity [5].

The laser intensity dependence of the visible PL bands indicates that in the upconversion process two laser photons are converted to one photon with larger frequency, while the behavior of the near-infrared emission is a simple downconversion process involving the absorption of only one laser photon as illustrated in Figure 21.

Three mechanisms may contribute for the enhancement of the antistokes emission due to the presence of the Si NPs. One mechanism may be the stepwise absorption of laser photons by the Si NPs with subsequent ET from double-excited Si NPs to Er^3+^ ions in the ground state. An other possible excitation pathway involves ET from two single-photon excited Si NPs to one Er^3+^ ion initially in the ground state. The third possibility is the energy transfer from single Si NPs to erbium ions already excited by the incident laser to level ^4^I_11/2_. These three processes should be considered because of the large density of Si NPs in the samples. However, at the present stage of the work, it is not possible to identify the actual mechanism contributing for the upconversion enhancement.

### 3.5. Third-Order Nonlinearity of PGO:Au Films

In order to exploit the large potential of germanate glasses for integrated optics, we developed a technique to produce PGO films containing silver NPs [26]. Radiofrequency cosputtering was used for deposition of noncrystalline films on silica substrates. The parameters of the fabrication process and the influence of the HT on the optical properties of the films were investigated. Good quality PGO films were produced and their nonlinear response was studied. In this section, we report on the third-order optical properties of PGO films containing gold NPs (PGO:Au). 

**Figure 22:**
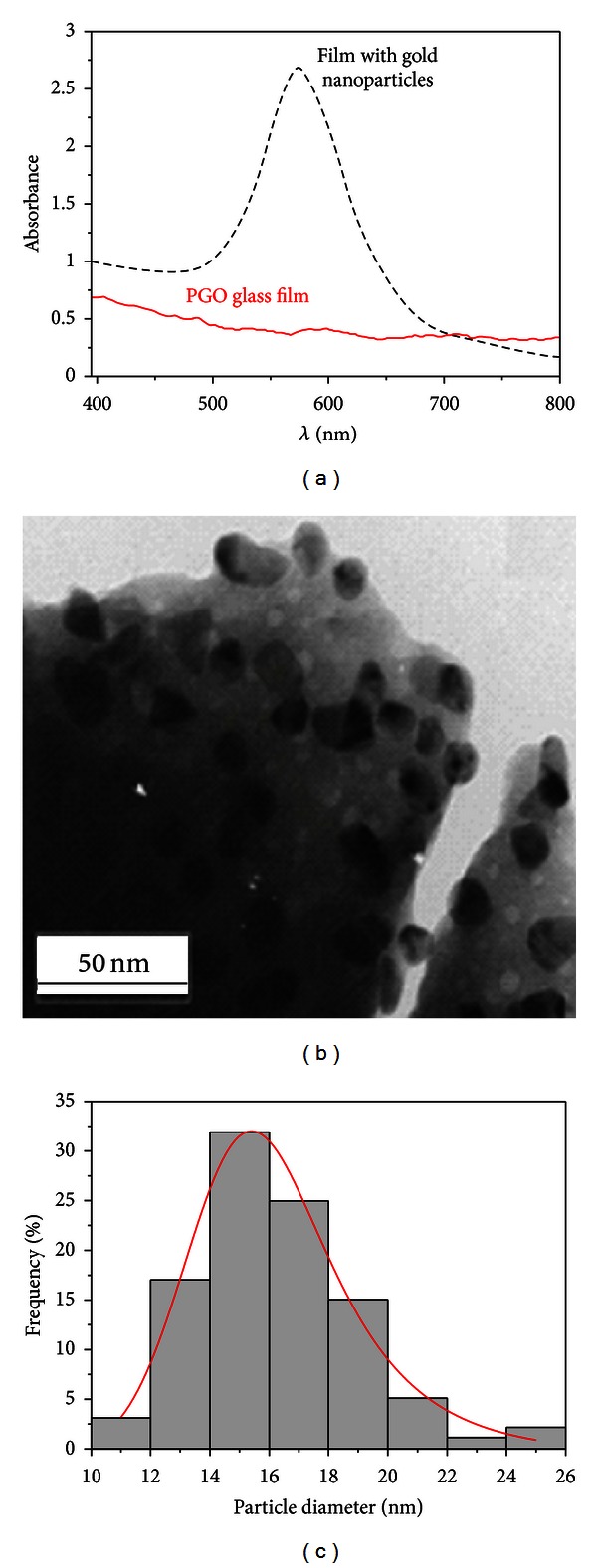
(a) Absorbance spectra of the PGO film with and without gold NPs at room temperature (films thickness: 1.1 *μ*m). (b) TEM image of the PGO:Au film. (c) Size distribution histogram of the gold NPs [5].

Figure 22(a) shows the absorption spectra of films without compensation of the Fresnel reflection contribution. Notice that while the spectrum of the glass film without gold NPs is almost flat along the whole wavelength range, a strong absorption band centered at *≈*575 nm (1.96 eV) is observed in the PGO: Au film due to the LSPR of the gold NPs.  Figure 22(b) shows a TEM image of the sample and Figure 22(c) presents the size distribution histogram of the NPs that have average diameter of 16 nm.

**Figure 23:**
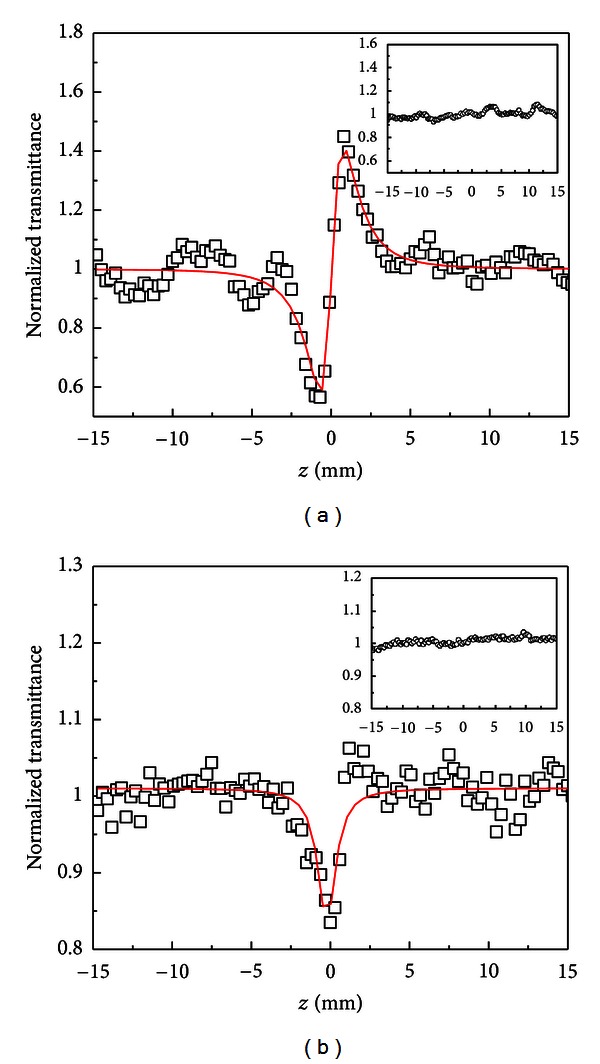
Z-Scan traces of the film with gold NPs at 532 nm (open squares). (a) Nonlinear refraction; (b) nonlinear absorption. The results for the film without gold (open circles) are shown in the insets. Laser intensity: 560 MW/cm^2^[27].

Figure 23 shows Z-scan profiles for the films with and without gold. The Z-scan technique allows measurements of the third-order susceptibility of materials [20]. From the *closed aperture* Z-scan profile, the amplitude and signal of the nonlinear refractive index *n*_2_ are determined. A typical Z-scan profile of the film with gold is shown in Figure 23(a) indicating a focusing nonlinearity (*n*_2_ > 0), while the inset in the figure shows the result for the film without gold NPs that has negligible nonlinearity.  Figure 23(b) shows an *open aperture* Z-scan profile for the film with gold NPs and the inset shows negligible nonlinear absorption for the film without gold. This measurement allows obtaining the nonlinear absorption coefficient *α*_2_. The solid lines represent the theoretical fitting to the experimental data based on the procedure introduced in [20].

The values of *n*_2_ = (6 ± 1) × 10^−10^ cm^2^/W measured at 532 nm and *α*_2_ = (1.7 ± 0.3) × 10^4^ cm/GW are attributed to the NPs contribution, since we did not observe nonlinear signals in the film without gold. Carbon disulfide (CS_2_), in a cell of 1 mm length, was used as standard calibration material with *n*_2_ = 3.1 × 10^−14^ cm^2^/W [27].

**Figure 24:**
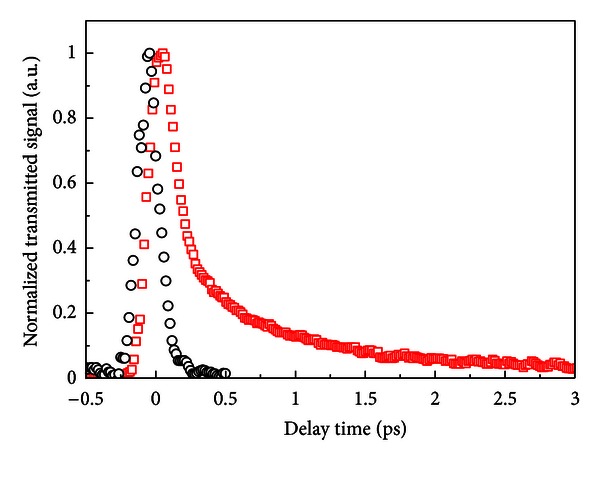
Normalized Kerr gate signal for CS_2_ (open squares) and for the film with gold NPs (open circles). Laser wavelength: 800 nm. Beams' intensities: *I*_pump_ = 342 MW/cm^2^ and *I*_probe_ = 45 MW/cm^2^. [27].

The nonlinear birefringence induced in a pump-probe experiment using a Kerr gate setup was also studied using a laser operating at 800 nm (pulses of 150 fs at 76 MHz).  Figure 24 shows the behavior of the normalized Kerr gate signal of film with gold NPs as a function of the delay time between the pump and probe pulses.

The signal corresponding to CS_2_, with two decay times: a fast decay of <50 fs and a slow one of *≈*2 ps, is also shown to illustrate the time resolution of the apparatus. The symmetric signal obtained with the PGO:Au film indicates that the nonlinearity is faster than the laser pulse duration. Also, by measuring the behavior of the probe beam signal as a function of the pump beam intensity, we obtained *n*_2_ = (10 ± 2) × 10^−13^ cm^2^/W. In addition, we investigated the transmittance of the film as a function of the laser intensity. No changes of the transmitted intensity was detected indicating that *α*_2_ was smaller than the sensitivity of the setup (<60 cm/GW).

The nonlinear experiments revealed ultrafast response of the film with gold NPs and enhanced values of the nonlinear refractive index at 532 and 800 nm due to the presence of gold NPs. A large nonlinear absorption coefficient was measured at 532 nm indicating the possible use of the films for optical limiting in the picosecond regime. The HT of the films improved the figure of merit for all-optical switching in comparison with the results obtained with the films without gold NPs by two orders of magnitude at 800 nm [6]. 

## 4. Conclusion

In conclusion, considerable progress has been made over the last decade on obtaining efficient luminescent composites from germanate and tellurite glasses doped with rare-earth ions containing metallic and semiconductor nanoparticles. The work has led to the production of good optical quality samples that present appropriate characteristics for photoluminescence-based devices. The possibilities to extend the work towards development of nonlinear optical composites are large, and we expect to be successful obtaining efficient materials for nonlinear photonics in the future.

## Figures and Tables

**Table 1:** Composition and parameters used for production of the glass samples. M/A refer to melting/annealing.

Sample	Glass composition (wt.%)	Dopants (wt.%)	M/A temperature (°C)	M/A time (h)
PGO-Er/Yb:Ag	40.3GeO_2_-59.7PbO	0.5Er_2_O_3_-3.0Yb_2_O_3_-1.0AgNO_3_	1200/420	1/2
PGO-Eu:Ag	40.3GeO_2_-59.7PbO	0.5Eu_2_O_3_-3.0AgNO_3_	1200/420	1/3
BGO-Eu:Au	58.4GeO_2_-41.6Bi_2_O_3_	0.5Eu_2_O_3_-3.0Au_2_O_3_	1100/420	1/3
BGO-Er:Si	58.4GeO_2_-41.6Bi_2_O_3_	0.5Er_2_O_3_-0.2Si nanopowder	1100/420	1/3
TZO-Er:Ag	85.0TeO_2_-15.0ZnO	0.5Tm_2_O_3_-2.0AgNO_3_	800/325	20/2
TWB-Er:Ag	54.5TeO_2_-22.6WO_3_-22.7Bi_2_O_3_	1.0Er_2_O_3_-2.0AgNO_3_	760/360	45/1
